# Mouse embryo CoCoPUTs: novel murine transcriptomic-weighted usage website featuring multiple strains, tissues, and stages

**DOI:** 10.1186/s12859-024-05906-3

**Published:** 2024-09-06

**Authors:** Sarah E. Fumagalli, Sean Smith, Tigran Ghazanchyan, Douglas Meyer, Rahul Paul, Collin Campbell, Luis Santana-Quintero, Anton Golikov, Juan Ibla, Haim Bar, Anton A. Komar, Ryan C. Hunt, Brian Lin, Michael DiCuccio, Chava Kimchi-Sarfaty

**Affiliations:** 1 Hemostasis Branch 1, Division of Hemostasis, Office of Plasma Protein Therapeutics CMC, Office of Therapeutic Products, Center for Biologics Evaluation and Research (CBER), US Food and Drug Administration (FDA), Silver Spring, MD USA; 2 High-performance Integrated Virtual Environment (HIVE), Office of Biostatistics and Pharmacovigilance (OBPV), Center for Biologics Evaluation and Research (CBER), US Food and Drug Administration (FDA), Silver Spring, MD USA; 3 Department of Anesthesiology, Critical Care and Pain Medicine, Boston Children’s Hospital and Harvard Medical School, Boston, MA USA; 4 Department of Statistics, University of Connecticut, Storrs, CT USA; 5 Department of Biological, Geological and Environmental Sciences, Center for Gene Regulation in Health and Disease, Cleveland State University, Cleveland, OH USA; 6 Rockville, USA

**Keywords:** Transcriptomic-weighted embryo data, Codon usage, Mouse embryo CoCoPUTs, Codon pair usage, GC content, Dinucleotide usage, Junction dinucleotide usage, Tissue-specific, Embryonic day, Theiler stage

## Abstract

Mouse (*Mus musculus*) models have been heavily utilized in developmental biology research to understand mammalian embryonic development, as mice share many genetic, physiological, and developmental characteristics with humans. New explorations into the integration of temporal (stage-specific) and transcriptional (tissue-specific) data have expanded our knowledge of mouse embryo tissue-specific gene functions. To better understand the substantial impact of synonymous mutational variations in the cell-state-specific transcriptome on a tissue’s codon and codon pair usage landscape, we have established a novel resource—Mouse Embryo Codon and Codon Pair Usage Tables (Mouse Embryo CoCoPUTs). This webpage not only offers codon and codon pair usage, but also GC, dinucleotide, and junction dinucleotide usage, encompassing four strains, 15 murine embryonic tissue groups, 18 Theiler stages, and 26 embryonic days. Here, we leverage Mouse Embryo CoCoPUTs and employ the use of heatmaps to depict usage changes over time and a comparison to human usage for each strain and embryonic time point, highlighting unique differences and similarities. The usage similarities found between mouse and human central nervous system data highlight the translation for projects leveraging mouse models. Data for this analysis can be directly retrieved from Mouse Embryo CoCoPUTs. This cutting-edge resource plays a crucial role in deciphering the complex interplay between usage patterns and embryonic development, offering valuable insights into variation across diverse tissues, strains, and stages. Its applications extend across multiple domains, with notable advantages for biotherapeutic development, where optimizing codon usage can enhance protein expression; one can compare strains, tissues, and mouse embryonic stages in one query. Additionally, Mouse Embryo CoCoPUTs holds great potential in the field of tissue-specific genetic engineering, providing insights for tailoring gene expression to specific tissues for targeted interventions. Furthermore, this resource may enhance our understanding of the nuanced connections between usage biases and tissue-specific gene function, contributing to the development of more accurate predictive models for genetic disorders.

**Supplementary Information:**

The online version contains supplementary material available at 10.1186/s12859-024-05906-3.

## Background

Mouse embryology and tissue-specific transcriptomics stand at the forefront of developmental biology, providing invaluable insights into the intricate processes that govern embryonic development and tissue differentiation [1–3]. *Mus musculus* has become an essential organism for studying embryogenesis due to its genetic proximity to humans, short reproductive cycles, and well-characterized genome. Investigating the dynamic changes in gene expression during different stages of mouse embryonic development offers a comprehensive view of the molecular events driving tissue specification [4–8].

Tissue-specific transcriptomics has given researchers a unique opportunity to dive deeper into gene expression profiles, unraveling the spatiotemporal intricacies of embryonic development. Recently, tissue-specific transcriptomics has revealed many normal and disease-specific gene expression associations. Joining transcriptomics and epigenetics helped identify several neuronal repressors enriched during early development [9]. Zhao et al. (2022) collected mouse embryo gut tissue samples spanning E9.5 to E15.5 to create a spatiotemporal transcriptome map, revealing critical developmental decisions are regulated by mesenchymal-epithelial interactions [10]. The integration of mouse embryology with tissue-specific transcriptomics not only advances our understanding of normal development but also unveils potential links to congenital disorders, paving the way for innovative therapeutic strategies and precision medicine approaches [11, 12]. Although the biological processes of developmental embryonic stages are well-established, the exact factors dictating genetic programming during development and the impact of variations in the cell-state-specific transcriptome on healthy tissue development in prenatal stages remain elusive.

To help close this gap and aid further embryology research, we combined temporal murine tissue-specific transcriptomics and gene-specific usage data from a collection of bulk RNA-seq mouse embryo samples sourced from three archives, more than 80 published articles, and more than 20,000 associated RefSeq Select gene transcripts [13]. Following different types of usage bias, such as GC, dinucleotide, junction dinucleotide, codon, and codon pair, over time have shown useful in distinguishing between species, variants, and strains [14–18], as a rationale for transcript design [19], for optimization and deoptimization projects, and many others [20–23].

GC content in the third position of a codon (GC3%) has been shown to be an important influence on gene expression patterns associated with distinct stages of development [24, 25]. Moreover, the utilization of codons ending with CG dinucleotides, especially in genes containing CG islands, are essential for proper development [26]. Fornasiero and Rizzoli [27] found predominantly A- or U-ending codons in cancerous tissue over control across 75 datasets and 40 pathologies, with a direct causal link to transcript production [27].

Here, we generated a new publicly accessible resource, Mouse Embryo CoCoPUTs website [13], to provide the median transcriptomic-weighted usage values for 1,381 mouse embryo samples. Mouse Embryo CoCoPUTs provides users access to GC, dinucleotide, junction dinucleotide, codon, and codon pair usage types that are easily downloadable and automatically displayed as tables, bar graphs, and heatmaps for each strain and embryonic stage of choice [13]. This webpage provides tissue- and stage-specific usage data for strain C57BL/6 (the most widely used inbred strain), the Jackson Laboratory strains C57BL/6N and C57BL/6J, and an outbred strain CD-1, which can be easily compared to usage values of a variety of organisms [28], human tissues [28], and cancers [29]. This tool can be used to characterize differences in usage patterns between disease and non-disease genes [30–33] and genes that have been identified as potential druggable targets [34, 35]. Similar webpages like CoCoPUTs [36], TissueCoCoPUTs [28], and CancerCoCoPUTs [29] have proven to be useful resources in identifying usage differences among organisms, human tissue types, and cancer types, respectively.

## Construction and content

### Data collection and sample selection

Data was collected from NCBI Sequence Read Archive [37], Mouse Genome Informatics RNA-Seq and Microarray Experiment Search database [38], and literature search was utilized to identify bulk RNA-seq mouse embryo samples from one of four strains: C57BL/6, C57BL/6J, C57BL/6N, and CD-1 (Additional File 1 and 2). Sex included male, female, and pooled. Cross-strain or genetically modified samples were removed, as well as samples receiving drug treatments or specialized diets. Samples from cultured cells were not included. Fastq files for 1,381 samples across 84 publications and projects were downloaded from NCBI [37]. Downloaded data comprised of single- and paired-end reads sequenced on AB SOLiD, Helicos Heliscope, or Illumina sequencers. We calculated transcript per million values using DRAGEN v3.7.5 [39] with the following parameters:

--enable-duplicate-marking true --enable-rna true --enable-rna-quantification true --annotation-file GCF_000001635.27_GRCm39_genomic.gtf.

Reads were aligned to the GRCm39 mouse reference genome and annotation file based on strain C57BL/6J (mm39, GCF_000001635.27) obtained from NCBI [37]. To automate DRAGEN analysis, processing was performed on the High-performance Integrated Virtual Environment [40]. We removed 22 pseudogenes from the 21,210 RefSeq and transcripts per million data that were identified via the C57BL/6NJ pseudogenes from the website *Mouse Strains Pseudogenes* (ADAM1A, ADAM1B, ADAM5, ATP6AP1L, FADS2B, FER1L4, GGNBP1, GLRA4, GLYCAM1, GUCY1B2, LY6G6E, MFSD13B, MPTX1, NPY6R, OFCC1, SERHL, SMPD5, TDH, TMCO5B, TMEM198B, TRPC2, and UOX) [41]. This resulted in 20,903 genes for further analysis.

### Transcriptome-weighted usage calculations

Gene-specific dinucleotide, junction dinucleotide, codon, and codon pair counts were prepared as matrices. Each value represents the number of times a particular codon (for example) appears in the coding sequence of a specific gene’s primary transcript. A median sample was constructed by computing the median transcript per million across all samples for a particular embryonic tissue type and stage. Using dot multiplication to multiply the sample gene counts (transcripts per million table) and the gene usage values results in the transcriptome-weighted dinucleotide, junction dinucleotide, codon, or codon pair usage values. This calculation was applied to four embryonic strains (C57BL/6, C57BL/6J, C57BL/6N, and CD-1), 15 tissue types, 18 Theiler stages (TS), and 26 embryonic days (Table 1). Dinucleotide, junction dinucleotide, and codon values were then normalized to one thousand and codon pair usage to one million. The metadata of the embryonic samples can be found in Additional File 2. The 15 tissue categories discussed here and found on the Mouse Embryo CoCoPUTs are a generalization of many highly specific tissues listed in Additional File 3 under the “Mouse Embryo” tab.

**Table 1:** Mouse Embryo CoCoPUTs sample data overview

**Strains**	C57BL/6	C57BL/6J	C57BL/6N	CD-1
**Tissues**	Central Nervous System	Eye	Face Head & Neck	Gonad
	Heart	Kidney	Limbs	Liver
	Lung and Bronchus	Pancreas	Small & Large Intestine	Spleen
	Stomach	Thymus	Whole Embryo	
**Embryonic Day (E)**	E6.25	E6.5	E7	E7.5
	E8	E8.5	E9	E9.5
	E10	E10.5	E11	E11.5
	E12	E12.5	E13	E13.5
	E14	E14.5	E15	E15.5
	E16	E16.5	E17	E17.5
	E18	E18.5		
**Theiler Stage (TS)**	TS00*	TS10	TS12	TS13
	TS14	TS15	TS16	TS17
	TS18	TS19	TS20	TS21
	TS22	TS23	TS24	TS25
	TS26			

* TS00 captures all samples that were not associated with a true Theiler Stage

#### Mouse Embryo CoCoPUTs

In our research, we developed a dashboard using Shiny (version 1.7.5) within the R programming environment (version 4.1.3), aiming to provide visual insights and facilitate interactive data exploration in a manner akin to the CoCoPUTs [36] methodology, all built upon the HIVE [40]. HIVE, noted for its efficiency in handling, analyzing, and storing vast datasets, serves as the backbone for our application's data management capabilities.

Shiny [0.2] has become a cornerstone in the R community for crafting dynamic web applications and dashboards. It uniquely integrates data analysis, visualization, and user interaction directly within the R ecosystem. Our dashboard's design incorporates a variety of user interface elements, including dropdown menus, tabs, buttons, and interactive plots, to foster an engaging user experience. On the server side, we employ functions to perform computations, generate visualizations, and dynamically update the user interface (UI) in response to user inputs. These server-side functions are crucial for seamlessly handling the intricate backend processes underlying the dashboard’s operational logic.

The adoption of Shiny's reactive programming model is instrumental in our dashboard, enabling a fluid dialogue between the UI and server-side components. This model ensures that the dashboard can respond to user interactions with real-time updates. Moreover, we have enhanced the dashboard's visual aesthetics and functionality by incorporating custom CSS and HTML, alongside integrating external libraries such as Plotly. This integration not only elevates the dashboard’s design but also enriches its interactivity, offering users sophisticated, interactive plots that enrich their data exploration experience.

### Example data analysis

The example data analysis discussed in this paper resulted from downloading the central nervous system samples across all TS from Mouse Embryo CoCoPUTs [13]. Heatmaps were developed to highlight the differences and similarities in GC, dinucleotide, junction dinucleotide, codon, and codon pair usage over each TS. We also compared the embryonic usage data to human usage data following the ratio of mouse embryo by human usage. We downloaded human tissue-specific data from TissueCoCoPUTs [13] and sorted the tissues into the more general categories used for the embryonic tissues (Additional File 3). Heatmaps were used to demonstrate biases and changes to usage and were created using Python (version 3.10.4) library Seaborn [42] and the graphics environment Matplotlib [43].

Significance was calculated between strains at each time point for a particular usage type (Additional File 4). For example, we tested whether C57BL/6 AAG (Arg) codon distribution was significantly different than C57BL/6J AAG codon distribution during the embryonic stage TS20. These comparisons were calculated per strain per TS for each usage type. We also compared TS within each strain for each usage type. We used Python’s (version 3.8) SciPy library [44], and Pandas to run a two-sided Mann–Whitney U test to find the raw *p*-values for each of the tests performed. Applying the statsmodels multipletests package (version 0.15.0), we adjusted the *p*-values using the Bonferroni correction (0.05/N), where significance is dependent on the number of tests performed (N). If the raw *p*-value is less than the adjusted threshold, the null hypothesis is rejected. The magnitude of the *p*-value, effect size, is determined by calculating Cohen’s D, with the expectation of unequal variances (Additional File 4). Effect sizes can be ‘very small’ (0—0.1), ‘small’ (0.2—0.35), ‘medium’ (0.36—0.65), ‘large’ (0.66—0.9), and ‘very large’ (> 1).

## Utility and discussion

### User walkthrough of Mouse Embryo CoCoPUTs

**Fig. 1:**
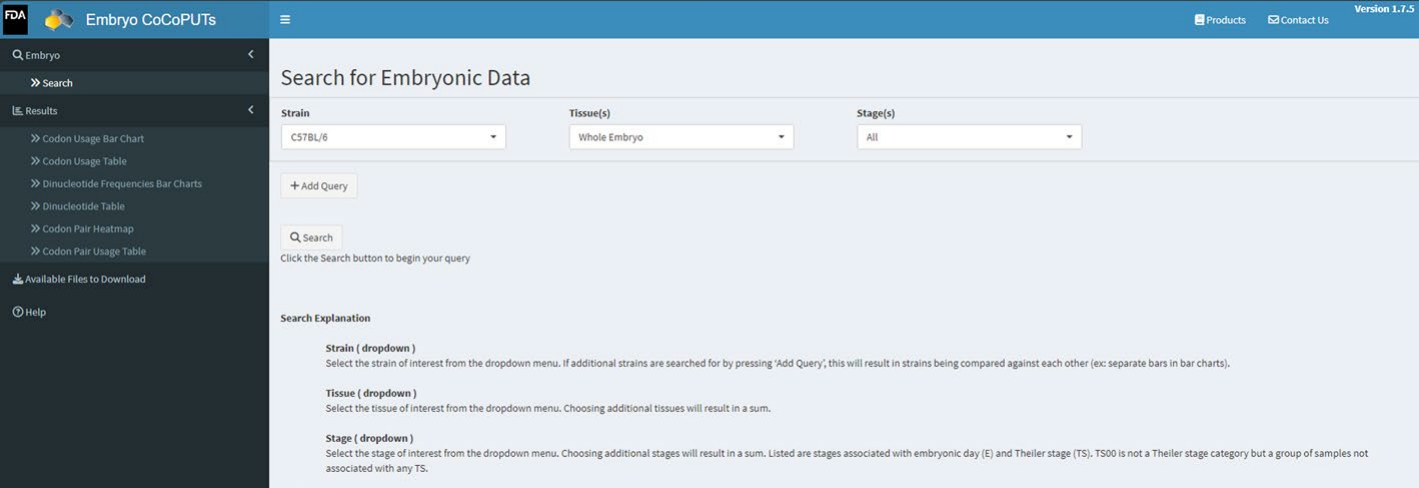
Mouse Embryo CoCoPUTs webpage interface. Users can search data tables by selecting one of four strains, one or more tissues, and one or more stages. Multiple queries produce a comparison under each Results tab. Files can be downloaded individually or as a package within the Results tabs and under the ‘Additional Files to Download’ tab

Mouse Embryo CoCoPUTs is the first website to provide easily accessible transcriptomic-weighted murine embryo GC, dinucleotide, junction dinucleotide, codon, and codon pair usage data for a variety of embryonic strains, tissues, and stages [13]. Mouse Embryo CoCoPUTs makes it easy to compare embryonic usage data by either downloading the data for local use or by leveraging the tables, bar graphs, and heatmaps that are automatically generated upon search inquiries (Fig. 1).

**Fig. 2:**
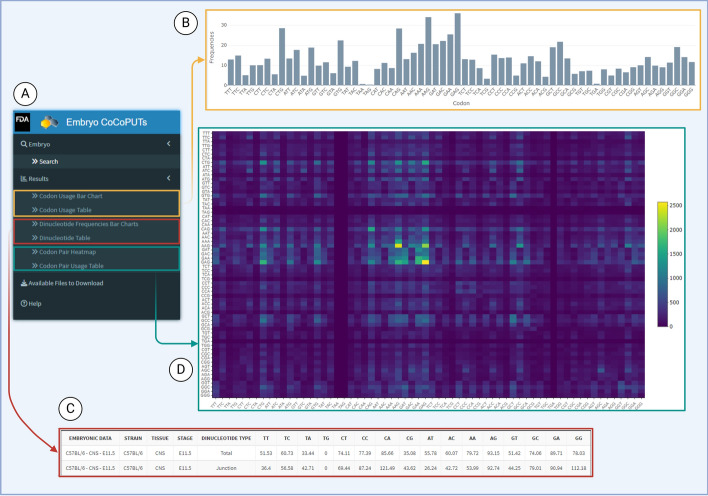
Mouse Embryo CoCoPUTs User Search Results. After selecting one or more strains, tissues, and stages, results are populated under tabs located on the left side of the search screen seen in Panel A. Codon Usage Bar Chart (Panel B) and Codon Usage Table display the codon usage and GC content as bar graphs and tables. Dinucleotide Frequencies Bar Charts and Dinucleotide Table (Panel C) display dinucleotide and junction dinucleotide usage as bar graphs and tables. Codon Pair Heatmap (Panel D) and Codon Pair Usage Table provide the codon pair usage as a downloadable heatmap or table

Once a user has input strain(s), tissue(s), and stage(s) into the Mouse Embryo CoCoPUTs query, results will quickly populate under the tabs on the left-hand side as seen in Fig. 1, also shown in Panel A of Fig. 2 [13]. Tabs ‘Codon Usage Bar Chart’ and ‘Dinucleotide Frequency Bar Charts’ provide the user with bar charts representing transcriptome-weighted codon usage (Panel B), dinucleotide usage, junction dinucleotide usage, and GC content. Tabs ‘Codon Usage Table’, ‘Dinucleotide Table’, and ‘Codon Pair Usage Table’ show the median usage for each query in easily downloadable tables that look like table displayed in Panel C of Fig. 2. Codon pair results were generated and can be found under the tab ‘Codon Pair Heatmap’ (Panel D). For graph clarity, it can be downloaded as a PNG or PDF. Query, result file descriptions, and a walk-through example can be found in the Help file (Fig. 2 Panel A at bottom).

To demonstrate the utility of the Mouse Embryo CoCoPUTs, we present an example of tissue-specific relationships across Theiler stages (TS) using the central nervous system (CNS) as a model tissue. First, we use heatmaps to provide a visualization of usage differences and similarities over time per mouse strain, and next, we compare mouse embryo to human CNS usage over time.

### G/C heavy usage highlights embryonic mouse strain specific differences across Theiler stages

**Fig. 3:**
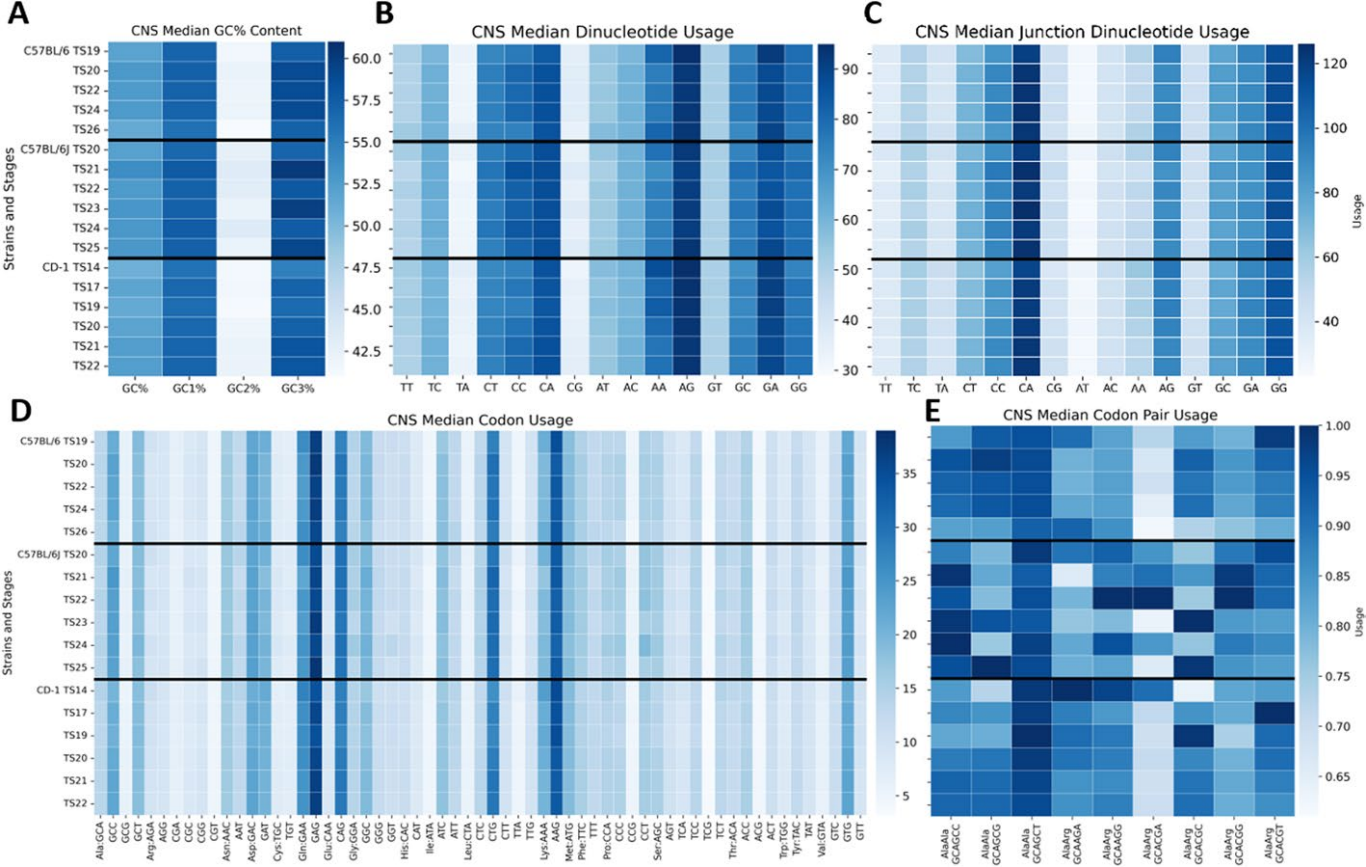
Mouse Embryo Usage over Theiler stage heatmaps for central nervous system genes. Each heatmap is subdivided on the y-axis by strain and Theiler stage. The darker the blue, the higher the usage. Panel A shows all strains have the least GC content in the second codon position. There is a strong preference for dinucleotide AG (Panel B) and junction dinucleotide CA (Panel C) for all strains. Codon usages are similar across all strains, leading with GAG (Gln) and AAG (Arg) (Panel D). Panel E describes the codon pair usage (scaled) for all synonymous Alanine:Alanine (AlaAla) and Alanine:Arginine (AlaArg) codon pairs, revealing very little variation in usage across strains than any other type of usage

We were interested in investigating how different usages changed over time for stains C57BL/6, C57BL/6J, and CD-1 within our CNS Mouse Embryo CoCoPUTs temporal dataset. Figure 3 lists each strain and its stages on the y-axis and the type of usage along the x-axis (GC content, dinucleotide, junction dinucleotide, codon, and codon pair usage are respectively associated with Panels A, B, C, D, and E).

## Key findings

- For all strains, GC usage is greatest in the third codon position (GC3%) and least in the second codon position (GC2%).
- None of the strains used the TG dinucleotide or junction dinucleotide (removed from Fig. 3 Panels B and C).
- All strains prefer consecutive nucleotides C and A between codons as junction dinucleotides, suggesting within codon positioning may play an important role in development.
- Codons GAG (Glu), CAG (Gln), CTG (Leu), AAG (Lys), and GTG (Val) are frequently used across all strains and TS.
- Codon pair usage was least variable between strains and stages, suggesting that the surrounding codon environment may be one variable that is most consistent between strains. Codon pair GCACGA (AlaArg) highlights the most divergence in usage for C57BL/6J compared to C57BL/6 and CD-1. Conversely, codon pair GCAGCT (AlaAla) is the most stable across strains.

The mouse embryo usage heatmaps overall highlight similarities in different types of usages between strains across stages of development. The lack of unique differences between these usage biases across murine strains suggest that similar gene expression patterns underlie the development of the CNS. Future studies may leverage this website to understand variation in other tissue types. To further distinguish whether unique CNS usage differences occur among strains through specific TS transitions, we generated heatmaps based on change in usage over time. By plotting the change over time, we can see more easily slight shifts in usage values.

**Fig. 4:**
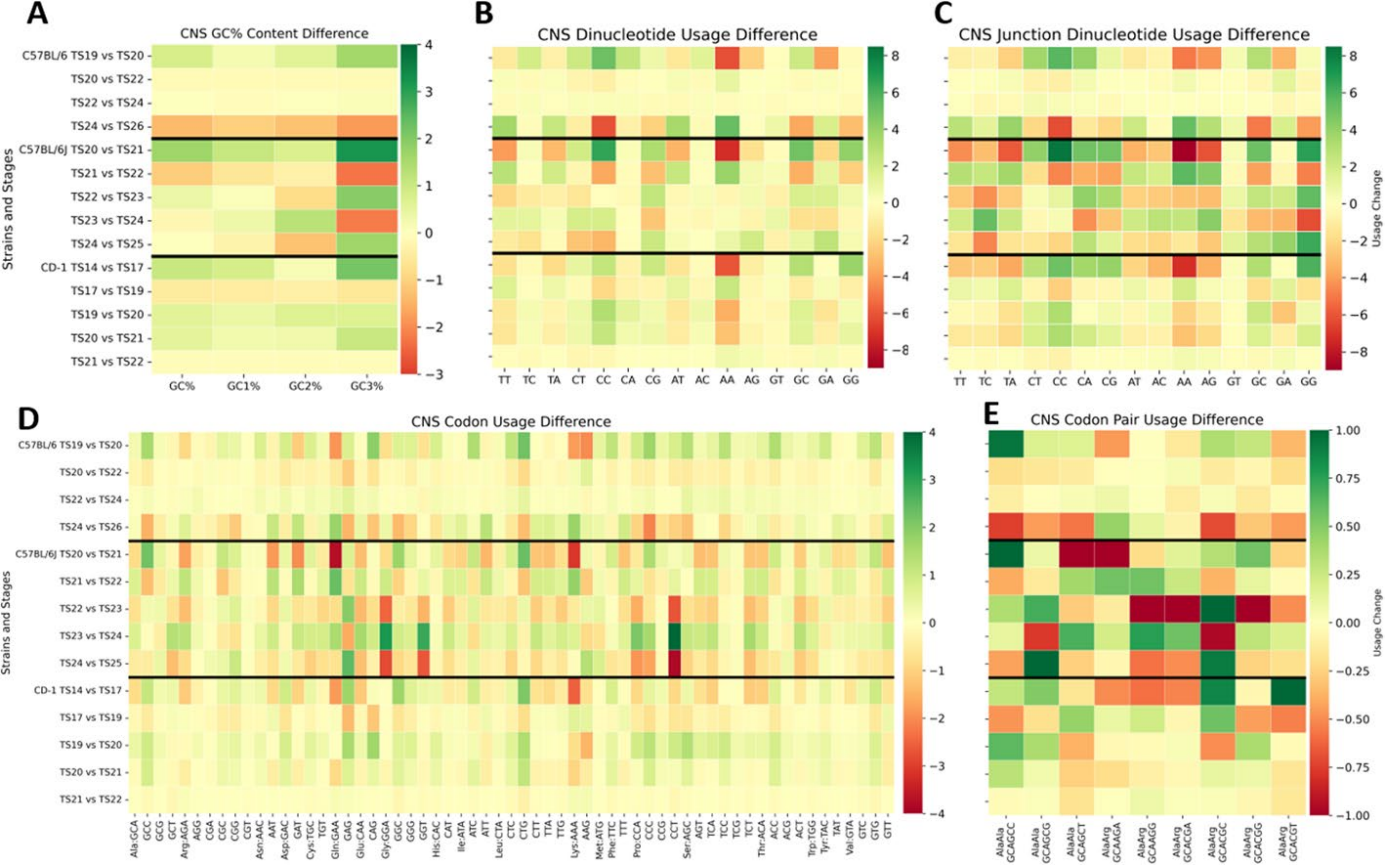
Difference over Theiler stage heatmaps for central nervous system genes reveal repeated direction reversals. Each heatmap is subdivided on the y-axis by strain and Theiler stage. Each row represents the change in usage from one stage to the next. Green represents an increase in usage, red is a decrease in usage, and yellow is centered on no change. Panel A shows the most drastic fluctuations in GC3 percent for C57BL/6J in comparison to C57BL/6 and CD-1 changes. Central nervous system dinucleotides (Panel B) tend to fluctuate less in general than junction dinucleotides (Panel C). Codon usage differences shown in Panel D reveal the majority of changes with the greatest magnitude are found within the Theiler stages of C57BL/6J. Panel E describes the codon pair usage difference (scaled) for all synonymous Alanine:Alanine (AlaAla) and Alanine:Arginine (AlaArg) codon pairs. This heatmap shows many small changes in codon pair usage for each of the strains over time

Figure 4 consists of five panels depicting usage change over time for each strain as one moves down the y-axis (GC content, dinucleotide, junction dinucleotide, codon, and codon pair usage are respectively associated with Panels A, B, C, D, and E). Green represents an increased change in usage (for example, from TS19 to TS20), red represents a decreased change in usage, and yellow is centered on zero to represent no change from stage to stage.

## Key findings

- Across all usage types, C57BL/6J CNS usage fluctuates more often than C57BL/6 and CD-1.
- C57BL/6 completely reverses usage direction for CC and AA dinucleotides and junction dinucleotides.
- Dinucleotide AC and junction dinucleotide GT were most consistent over time for all strains.
- A and T leading junction dinucleotides tend to increase as C and G leading tend to decrease (most obvious trend in C57BL/6J.
- Codon GAG (Glu) fluctuates more often than most other codons across all strains. At the end of TS22, C57BL/6 shows decline, while C57BL/6J shows an increase.
- Synonymous codons from amino acids glutamine, glutamic acid, glycine, and proline (G and C leading) show the most dramatic changes over time.
- C57BL/6 codon pair usage changes the most in the first and last TS, C57BL/6J is more uniform in its fluctuations over time, and CD-1 decreases activity as time progresses.

To identify usage comparisons that are statistically significant and their magnitudes for both within and between strains, we used the two-sided Mann–Whitney U test (Bonferroni corrected *p*-value $\ge$ 0.05) and Cohen’s D (> 1 valued results discussed here – see Additional File 4 for all results).

## Key findings

- C57BL/6 GC1, GC2, and GC3 content showed all TS comparisons were significantly different with very large Cohen’s D values (> > 1).
- Significant difference was found for C57BL/6 GC and GG dinucleotides and junction dinucleotides between TS20 and TS26.
- Codon usage for CD-1 was found significant between TS17, TS21, and TS22 for codon GGA (Gly).
- Several usage comparisons between C57BL/6 and CD-1 revealed a significant difference. The most notable embryonic time periods were TS20 and TS22, potentially leading to differences during development at these time points.

### Human versus mouse embryonic central nervous system usage change across Theiler stages for central nervous system samples

Studies have previously demonstrated that mouse and human brain tissues have selectively conserved codon usage across evolutionary development for CNS-specific genes [45]. Using Mouse Embryo CoCoPUTs and our previously generated website for human tissue-specific data from the TissueCoCoPUTs [28], we further evaluated similarities and differences among various usage metrics. We leveraged TissueCoCoPUTs and extracted human tissue usage data to match the analogous CNS categories used for the embryonic tissues (Additional File 3). We generated heatmaps, comparing mouse embryo usage to human usage, whereby if mouse usage is greater than human, the results will be greater than one (blue), and if human usage is greater, the result will be less than one (purple).

**Fig. 5:**
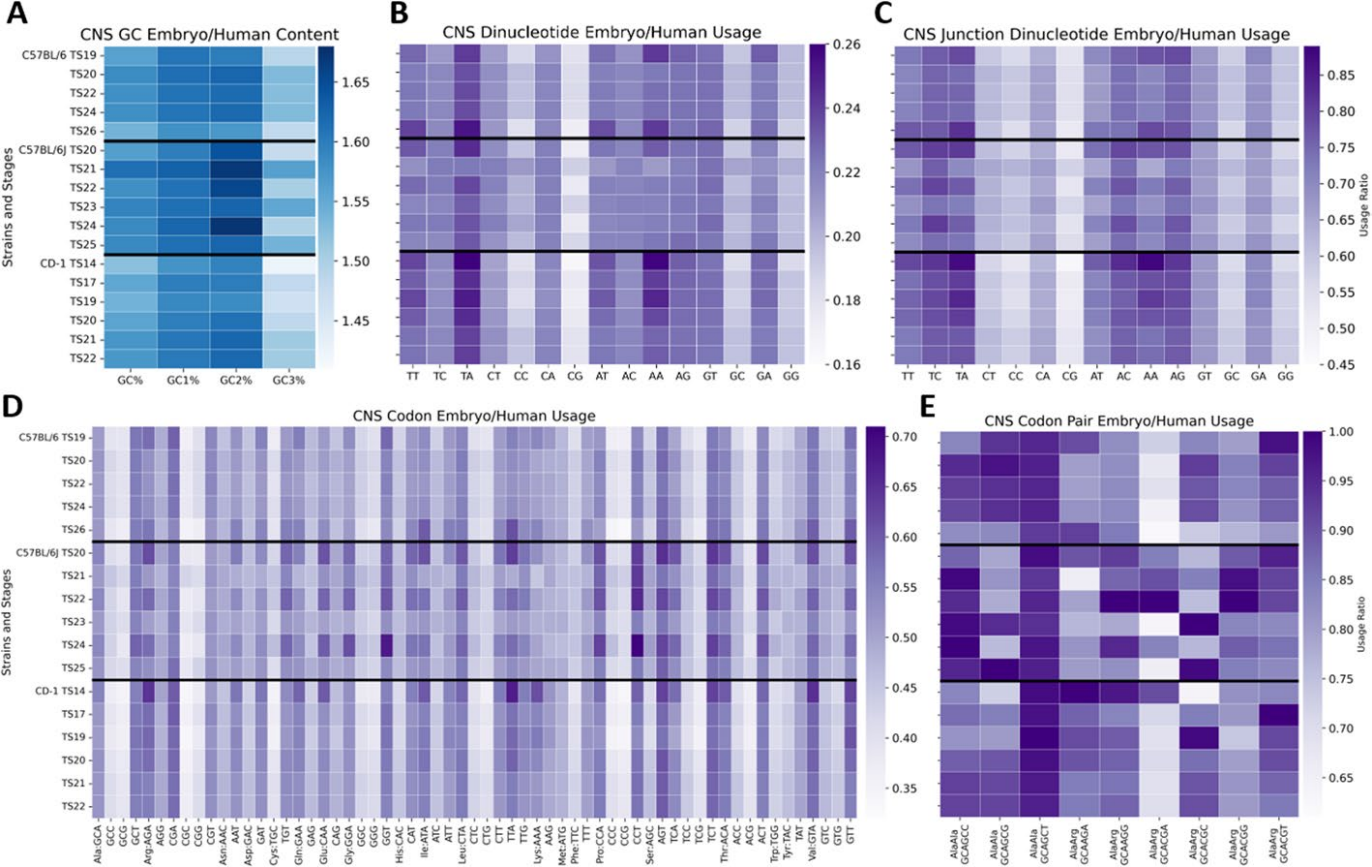
Mouse Embryo vs Human Usage over Theiler stages for central nervous system genes. Each heatmap is subdivided on the y-axis by strain and Theiler stage. If a heatmap is blue, embryo usage outweighs human usage (> 1). If the heatmap is purple, human usage outweighs embryo usage (< 1). Panel A shows all strains have greater GC content than central nervous system human usage. Panel B usage values are all less than one signifying that human dinucleotide usage is greater than embryo usage, especially TA and CA usage. Junction dinucleotides that lead with T or A nucleotides show the biggest difference between human and embryo usage (Panel C). Codon usages are similar across all strains, with C57BL/6J most skewed away from human usage (Panel D). Panel E describes the codon pair usage (scaled) for all synonymous Alanine:Alanine (AlaAla) and Alanine:Arginine (AlaArg) codon pairs. This heatmap shows a dramatic increase in human usage for GCAGCT (AlaAla)

Figure 5 shows the mouse embryo-human ratio for different types of usage across each strain and its TS for CNS genes (GC content, dinucleotide, junction dinucleotide, codon, and codon pair usage are respectively associated with Panels A, B, C, D, and E).

## Key findings

- Interestingly, human outweighed mouse embryo in all CNS usage categories, except for GC content.
- The biggest mouse contribution comes from GC2 usage, suggesting that GC content may be more integral towards codon mouse development.
- Human dinucleotide, junction dinucleotide, codon, and especially codon pair usage is very similar to mouse embryo, supporting mouse embryo usage values as good experimental representatives independent of the strain for CNS genes.

It has been shown that dinucleotide CG may impact mouse embryology through the movement of transposable elements and site methylation [46]. Within the 5’ untranslated region of LINE1 transposon (L1) promotors, day 0 (d0) showed very little methylation of CG dinucleotide sites, but by day 21 CG sites were more than 80% methylated. CG usage peaks near YY1 transcription factor binding sites have been shown to potentially direct DNA methylation towards L1 promotors, reducing their impact during development [46]. Both human and mouse embryo show a strong preference for dinucleotide CA, suggesting the location of the dinucleotide, at a codon junction versus elsewhere, may influence its usage and how it changes over time. Dinucleotide CA may play a secondary role in controlling the translation rate throughout fetal development.

Other studies have demonstrated an underrepresentation of certain dinucleotides in genes associated with disease [40]. For example, genes associated with neurodegeneration were shown to have less than expected dinucleotide CG, GT, and TA usage, positive correlations with CC, CG, CT, GC, and GG, and negative correlations with AA, AT, GA, TA, and TT [47]. Alqahtani et al. (2021) speculated that neurodegeneration-associated genes may have originated from viruses that eventually gain functionality, since humans and viruses share underrepresented dinucleotides CG, TA, and GT. Suppression of these dinucleotides may contribute to selection pressure, degradation, and/or methylation and deamination [47]. Within our CNS data, we found similar expectations for the C57BL/6, C57BL/6J, and CD-1 mouse strains. The human CNS dinucleotide usage data revealed an increase for CG, TA, and GT dinucleotides, especially TA—as it is one of the preferred dinucleotides. Identification and location of dinucleotides may be of importance in search of characteristics shared between mouse embryo and human and their relation to congenital diseases. Mouse Embryo CoCoPUTs may provide aid to clinical researchers in need of mouse models with specific GC criteria or target specific information per stage as well as many other uses [13].

Synonymous codon usage, specifically leucine and arginine, has also been shown to reduce embryonic mouse cell proliferation but not affect stem cell pluripotency [48]. A decrease in the production of these synonymous codons is directly related to a decrease in protein translation. Leca et al. [49] revealed a neurodevelopmental phenotype produced via differential synonymous codon usage that dramatically altered protein production leading to homozygous lethality [49]. Future studies evaluating different mouse strains, especially developmental studies spanning multiple embryonic stages or strain targeting for pre-clinical testing of therapeutics, should be aware of the impact of these usage differences. These findings are critical for understanding the relationship between these usage types and embryonic development, and provide the necessary biological context for future studies looking to elucidate disease-gene expression relationships across development.

Mouse Embryo CoCoPUTs provides researchers with unique access to transcriptomic-weighted mouse embryo usage data that can be compared between strains, tissues, and stages [13]. Any deviations in patterns of usage preferences provided on website may be indicators of developmental abnormalities and may be useful guiding a generation of novel disease predictors. Limitations of this resource are its inability to run calculations within the website (i.e., average, variance), difficulty in making comparisons over several embryonic stages, and comparisons to other species. Future goals of the Mouse Embryo CoCoPUTs website are to implement new features to overcome these limitations, update regularly with new samples, and add useful comparison calculations like relative synonymous codon usage, and expected number of codons (Enc) and expected number of codon pairs (Encp) to facilitate broader species comparisons.

## Conclusion

Mouse Embryo CoCoPUTs, a novel resource, holds the potential to facilitate investigations into tissue-, stage-, or strain-specific biotherapeutic development, genetic engineering, and genetic disease prediction [13]. Here, we describe a tool that combines gene sequence data and murine tissue- and stage-specific gene counts to create transcriptomic-weighted GC, dinucleotide, junction dinucleotide, codon, and codon pair usage across murine strains, tissues, and stages. The homepage of Mouse Embryo CoCoPUTs gives the user the option to query the usage website via murine strain, tissue, and stage (Table 1), download original transcriptome-weighted usage files, and a ‘Help’ tab that describes each search feature, different usage results (heatmaps, bar graphs, and tables), and methods for related calculations. Mouse Embryo CoCoPUTs can be used to identify relationships among embryonic strains, stages, and human usage (13). Across various metrics of usage, mouse embryo exhibited unique patterns and similarities across different strains C57BL/6, C57BL/6J, and CD-1.

## Supplementary Information

Additional file1.

Additional file 2.

Additional file 3.

Additional file 4.

## Data Availability

The raw datasets generated and analyzed during the current study are available for download from Mouse Embryo CoCoPUTs.

## Abbreviations

TS: Theiler stage
E: Embryonic day
CoCoPUTs: Codon and codon pair usage tables
CNS: Central nervous system
