# Supplementary material for: Mouse embryo CoCoPUTs: novel murine transcriptomic-weighted usage website featuring multiple strains, tissues, and stages
Source: BMC Bioinformatics. 2024 Sep 6;25:294. doi: 10.1186/s12859-024-05906-3 (PMC11380194; doi:10.1186/s12859-024-05906-3)
Supplement: Supplementary file 1 — Additional file1. [file 12859_2024_5906_MOESM1_ESM.docx]

Additional Files

Additional File 1.xlsx

Title: Embryonic mouse metadata

Description: File lists each mouse embryo sample by SRA accession, age, structure, strain, BioSample, sequencer, read length, library prep, project ID, and reference.

Additional File 2.xlsx

Title: Embryonic mouse and human tissue categories

Description: File lists general and specific tissue types contributing to the central nervous system samples for human and mouse embryo.

Additional File 3.zip

Title: Significance testing within and between mouse embryo strains across Theiler stages

Description: Zip folder contains several xlsx files that list the Mann-Whitney U stat and Bonferroni adjusted p-value and Cohen’s D metric for usage comparisons (GC content, dinucleotide, junction dinucleotide, codon, and codon pair) at different Theiler stages for a given strain and between strains.
